# Risk factors of work-related upper extremity musculoskeletal disorders in male cameramen

**DOI:** 10.1186/s40557-014-0052-x

**Published:** 2015-01-24

**Authors:** Jung ho Kim, Byung seong Suh, Soo Geun Kim, Won sool Kim, You il Shon, Hee seung Son

**Affiliations:** Department of Occupational and Environmental Medicine, Kangbuk Samsung Medical Center of Sungkyunkwan University, Seoul, Republic of Korea

**Keywords:** Occupational diseases, Cameramen, Ergonomics, Physical burden, BMI, Exercise

## Abstract

**Objective:**

The aim of this study is to determine the risk factors related to upper extremities work-related musculoskeletal disorders (WRMSDs) in cameramen.

**Methods:**

A questionnaire survey was performed on 166 cameramen in a broadcasting station. The questionnaire consisted of questions on the general characteristics, the health behavior, work type and duration, physical burden, ergonomic posture, and musculoskeletal symptoms. Definition of musculoskeletal disorders was based on NIOSH criteria.

**Results:**

The positive rate of WRMSDs symptoms by parts of the body was turned out to be the highest in the shoulder (14.5%) and the lowest in arm and elbow (6%). Logistic regression analysis revealed that symptoms in the shoulders increased with BMI (OR = 3.62, 95% CI = 1.03-12.71), physical burden (OR = 9.29, 95% CI = 1.72-61.78 in the very hard group) and ergonomic factors (OR = 4.50, 95% CI = 1.03-19.68). Ergonomic factors were only related to the symptoms of hand and wrist (OR = 10.21, 95% CI = 1.02-102.20). WRMSDs symptoms, in the whole upper extremities, were higher in the 50 or older age group (OR = 5.86, 95% CI = 1.03-33.26), higher BMI group (OR = 3.26, 95% CI = 1.28-13.53), non-exercise group (OR = 2.37, 95% CI = 1.24-12.59), high physical burden group (OR = 7.6, 95% CI = 1.34-52.74), and high grade ergonomic risk group (OR = 4.82, 95% CI = 1.29-16.06).

**Conclusion:**

The most serious musculoskeletal disorders of male cameramen were shoulder pain. Ergonomic factors and physical burden were the most significant factors affecting WRMSDs in cameramen in this study. Cameramen should be educated to be able to improve the ergonomic occupational environment and to set up preventive measures against the risk factors during work.

## Introduction

Work-related musculoskeletal disorders (WRMSDs) are serious socioeconomic problems in modern society from two point of view. First, WRMSDs are one of the most common work-related diseases in developed countries. Second, WRMSDs are key factors for sick leave, which is common around the world [1].

WRMSDs are known to be very common among workers who exposed to various occupational hazards, such as awkward working postures, repetitive manual work or long duration of work, according to the type of work [2-5].

In a previous study, the tasks performed by the cameramen were simulated, based on the results of monitoring the field performance of these camera operators for a two-week period. The results clearly show that jobs performed by the cameramen working for television stations include very demanding physical tasks [6]. And, physically demanding workload was identified as a significant risk factor for musculoskeletal disorders in the other previous studies [7,8].

Cameramen are generally thought to be at risks that can cause WRMSDs, since their work requires carrying heavy objects on the shoulder as a part of their job, maintaining the same posture for a long time. Furthermore, they sometimes have to work several hours without taking break depending on their recording schedules, which are also known as one of the risk factors for WRMSDs [9,10]. Although there are no previous studies that have compared the prevalence of WRMSDs of cameramen to other jobs, cameramen are comparatively thought to be at risk of WRMSDs.

Risk factors of WRMSDs should be studied in conformity with the jobs, as working environment and ergonomic aspects vary depending on their work types and parts of the body they suffer from are totally different according to the parts they mainly use.

Cameramen are usually divided into two groups. The first group works with a portable camera that weighs about 10Kg or heavier. As all types of the portable camera are right-handed, they cannot change the shoulders during working. In the face of these facts their musculoskeletal disorders are mostly influenced by the weight and contact stress. The other form of work is to control a studio camera that is fixed on the ground. They do not get any weight or contact stress from their work. Instead of that, they have to keep their posture all the time during working hours. In both type of work, they usually raise their arm and shoulder for a long time. Comparatively, their lower extremities are ergonomically stable. And they can stretch or change their lower extremities position while they are working. In consideration of their working posture and contact stress from the heavy camera, upper extremities will get a more harmful influence than lower extremities. Thus, we only focused on the upper extremities in this study.

Since little research has been done to understand about their musculoskeletal problems and to investigate which factors are more related with the occurrence of WRMSDs in cameramen, we wanted to find out which general risk factors of WRMSDs are actually able to influence on the WRMSDs in cameramen.

## Materials and methods

As stated above, cameramen use two different type of camera. But, most of them change their role as per instruction and type of shooting. Thus, their work is actually combined and very hard to divide into two groups. So, we surveyed all cameramen on the active list regardless of their current or past position. In the light of latent period of musculoskeletal disorders, we excluded some cameramen whose period of continuous service is under 1 year. Taking medicine or physical therapy for musculoskeletal disorders is also able to conceal related symptoms. Thus, those people are also excluded.

A questionnaire survey was conducted on 189 cameramen who work in a broadcasting station in Korea. After excluding 3 female workers, 9 workers who had been employed for less than 1 year, and 11 cameramen who are on medication or treatment for upper extremity musculoskeletal disease or accident, and those with an incomplete response to the questionnaire, 166 male cameramen were finally selected for the study population.

### Questionnaire

All questionnaires that used in this study were self-administered. The first questionnaire investigates personal variables such as age, sex, marital status, height, weight, exercise, physical burden, housework hour, use of current medication, recent trauma or accident history. The level of physical burden is measured subjectively. They were asked to choose the level of physical fatigue when they are working. The level of physical burden is made up of 3 items, which are ‘None to moderate’ , ‘Hard’ , ’Very hard’. This variable was significantly related with the positive rate of upper extremity musculoskeletal symptoms in a previous study [11].

As cameramen tend to work very irregularly and change their working position very often, they were requested to answer the questions on the basis of the past 3 month average experience when they are confused to answer accurately.

Since it is not a cost-effective way to investigate all workers and their musculoskeletal symptoms, Ministry of Employment and Labor conducts the risk factor investigation by using questionnaire. We adopted the questionnaires that ask about musculoskeletal symptoms and ergonomic factors [12-14]. The symptom questionnaire consists of duration, frequency, and severity of the symptoms on each body parts [12]. And, it is designed to apply NIOSH (National Institute of Occupational Safety & Health) symptom criteria [13]. The questionnaire asks if they experienced any pain or discomfort during past 1 year, classifies them into high risk group if the pain continued longer than 1 week or occurred more than 1 time during 1 month.

We calculated the complaints rate of musculoskeletal symptoms on the basis of the questionnaire by characteristics of the subjects. Aching parts are comprised of Head, shoulders, arms/elbows, hands/wrists/fingers. And if a subject has more severe pain than moderate level on any of these parts for longer than 1 week, we categorized them as the high symptom complaints group. Continuous variables were converted to the ordinal scale according to distribution to make the model stable and ensure biological plausibility.

Finally, ergonomic risk factor check list from OSHA (Occupational Safety and Health Administration) Draft Ergonomics Standard was given to the subjects to judge their work posture and to calculate ergonomic risk scores [14]_._ This check list is a proactive survey form, which enables quick assessment of ergonomic risks. We used a section for upper extremities, which is made up of 19 scales. Every scale assigns a score depending on the exposure time spent for the specific posture in a day. All scores are calculated after applying weighted value.

### Selection of variables

Age was divided into 3 groups by 10-year intervals (30–39, 40–49, 50-). BMI (Body Mass Index) was obtained by their height and mass (kg). Subjects who work out regularly (over 40 minutes at a time, more than 3 times a week) are considered to be a regular exercise group. Tenure was calculated by month, and subjects were classified under 4 tenure groups by quartile. Working hours (hour) and break time (minute, exclude mealtime) were divided into 2 groups on the basis of the distribution. Physical burden was categorized into 3 scales (none to moderate, hard, very hard). When it comes to ergonomic evaluation, we classified subjects into low and high risk groups according to the total upper extremities score of the ergonomic check list. A score of 10 or above is considered as a high ergonomic risk group according to guidelines of OSHA [14].

### Statistical analysis

After examining the distribution of each variable to determine its normality, univariate analysis was conducted to evaluate the effect of each variable on WRMSDs symptoms. Chi-square for trend test and chi-square test were used to analyze categorical variables. To adjust for the effect of confounders and to understand reciprocal action, multiple logistic regressions were conducted. Statistical significance was considered at a level of P <0.05. The software program SPSS for Windows, version 19.0, (SPSS, Chicago, IL) was used for statistical analysis.

## Results

Table 1 shows general characteristics and information about age, BMI, tenure, marital status, working hours, break time, exercise status, house work frequency, level of physical burden, ergonomic risk scores, and constitution of high risk symptom groups for upper extremity musculoskeletal disorders of respondents.

**Table 1:** **General characteristics of the subjects (n = 166)**

		**Total**		**Symptom high risk group**		**Symptom low risk group**	
	**Group**	**Number (%)**	**Mean (SD)**	**Number (%)**	**Mean (SD)**	**Number (%)**	**Mean (SD)**
**Age (year)**			46.2(7.7)		47.6(8.4)		45.9(7.5)
**BMI (kg/m** ^2^ **)**			24.1(2.4)		24.8(2.4)		23.9(2.4)
**Tenure (month)**			202.8(116.0)		233(122.5)		194(113.4)
**Working hours (hour)**			9.4(5.5)		10.2(2.7)		9.2(2.4)
**Break time (minute)**			42.0(32.4)		36.3(39.7)		43.5(30.2)
**Marital status**	Yes	154(92.8)		31(91.2)		123(93.2)	
	No	10(6.0)		3(8.8)		7(5.3)	
**Exercise**	Yes^*^	69(41.6)		6(17.6)		63(48.1)	
	No	96(57.8)		28(82.4)		68(51.9)	
**House work (hour)**	≥1 hour	29(17.5)		30(88.2)		104(78.8)	
	< 1 hour	134(80.7)		3(8.8)		26(19.7)	
**Physical burden** ^**†**^	N to M^‡^	75(45.2)		10(29.4)		65(49.2)	
	Hard	70(42.2)		13(38.2)		57(43.2)	
	Very hard	21(12.7)		11(32.4)		10(7.6)	
**Ergonomics** ^**§**^	High risk	75(45.2)		24(70.6)		51(43.2)	
	Low risk	77(46.4)		10(29.4)		67(56.8)	
**Symptoms** ^**∥**^	High risk	34(20.5)					
	Low risk	132(79.5)					

* Exercise Yes: >3 times/week, >40 minutes/one time.

^†^Physical burden: level of physical fatigue when working.

^‡^None to moderate.

^§^Ergonomics: check list from OSHA Draft Ergonomics Standard, a score of 10 or above is high risk group.

^∥^Symptoms: experience of any pain or discomfort during past 1 year. Any pain continued longer than 1 week or occurred more than 1 time during 1 month are classified into high risk groups.

Mean age of the subjects was 46.2 years(SD 7.67 years) and more than 75% of respondents were over the age of 40 (Table 2). Mean length of employment in the broadcasting station was 202.8 months. 50.0% of respondents work longer than 9 hours a day on the average, 63.4% of respondents had 30 minute or less break time except lunch and dinner time. Most subjects were married (92.8%), 41.8% of the subjects worked out regularly.

**Table 2:** **High risk rate of upper extremities musculoskeletal symptoms by characteristics of subjects (n = 166)**

		**Subjects**	**Neck**	**Shoulder**	**Arm/elbow**	**Hand/wrist**	**Upp. ext**
**Total**		166(100)	13(7.8)	24(14.5)	10(6.0)	11(6.6)	34(20.5)
**Age (year)**	30-39	35(21.1)	5(14.3)	3(8.6)	2(5.7)	2(5.7)	5(14.3)
	40-49	66(39.8)	1(1.5)	9(13.6)	3(4.5)	4(6.1)	12(18.2)
	≥50	65(39.2)	7(10.8)	12(18.5)	5(7.7)	5(7.7)	17(26.2)
**BMI (kg/m** ^2^ **)**	<25	90(63.8)	4(4.4)	6(6.7)*	2(2.2)*	3(3.3)*	7(10.0)^†^
	≥25	51(36.2)	6(11.8)	14(27.5)	7(13.7)	7(13.7)	18(37.3)
**Tenure (month)**	12-117	46(27.7)	4(8.7)	5(10.9)	2(4.3)	4(8.7)	8(17.4)
	118-212	37(22.3)	1(2.7)	3(8.1)	1(2.7)	2(5.4)	4(10.8)
	213-324	44(26.5)	4(9.1)	6(13.6)	3(6.8)	3(6.8)	10(22.7)
	≥325	39(23.5)	4(10.3)	10(25.6)	4(10.3)	2(5.1)	12(30.8)
**Working**	<9	77(50.0)	4(4.4)	8(10.4)	3(3.9)	2(2.6)*	10(13.0)*
**Hours (hour)**	≥9	77(50.0)	6(11.8)	14(18.2)	7(9.1)	9(11.7)	22(28.6)
**Break time**	<30	80(54.2)	7(8.8)	13(14.4)	3(3.3)	4(4.4)	19(21.1)
**(minute)**	≥30	52(31.3)	6(9.5)	7(13.5)	4(7.7)	6(11.5)	10(19.2)
**Exercise** ^**‡**^	Yes	69(41.6)	4(5.8)	5(7.2)*	2(2.9)	3(4.3)	6(8.7)^†^
	No	96(57.8)	9(9.4)	19(19.8)	8(8.3)	8(8.3)	28(29.2)
**House work**	<1 hr/day	134(80.7)	8(6.4)	20(14.9)	8(6.0)	9(6.7)	30(22.4)
	≥1 hr/day	29(17.5)	4(10.5)	3(10.3)	1(3.4)	1(3.4)	3(10.3)
**Physical**	N to M^§^	75(45.2)	1(1.3)*	7(9.3)†	5(6.7)	2(2.7)*	10(13.3)^†^
**burden**	Hard	70(42.2)	7(10.0)	8(11.4)	3(4.3)	5(7.1)	13(18.6)
	Very hard	21(12.7)	5(23.8)	9(42.9)	2(9.5)	4(19.0)	11(52.4)
**Ergonomics** ^**∥**^	Low risk	78(51.3)	3(3.8)*	7(9.0)^†^	4(5.1)	2(2.6)*	11(14.1)^†^
	High risk	74(48.7)	10(13.5)	17(23.0)	6(8.1)	9(12.2)	23(31.1)

Chi-square test or Fisher’s exact test: Working hours, Break time, Exercise, House work, Ergonomics, BMI.

Chi-square test for trend : Age, Tenure, Physical burden.

*p <0.05 by chi-square test or chi-square test for trend, comparison between subgroups.

^†^p <0.01 by chi-square test or chi-square test for trend, comparison between subgroups.

^‡^Exercise Yes: >3 times/week, >40 minutes/one time.

^§^N to M: None to moderate.

^∥^Ergonomics: check list from OSHA Draft Ergonomics Standard, a score of 10 or above is high risk group.

Variables such as age, tenure, BMI, working hour, house work, physical burden, ergonomic risk were checked higher in symptom high risk group. On the other hand, break time, marital status, regular exercise group were higher in symptom low risk group.

As shown in Table 2, the positive rate of WRMSDs symptoms by parts of the body was turned out to be the highest in the shoulder (14.5%) and the lowest in the arm and elbow (6%). Factors that are found to be related to the symptoms of WRMSDs in Table 2 are BMI, working hours, exercise, physical burden and ergonomic factors. Those correlations vary in accordance with parts of the body. There was no significant difference according to age, tenure, break time, house work.

Logistic regression analysis revealed that symptoms in the shoulders increased with BMI (OR = 3.62, 95% CI = 1.03-12.71), physical burden (OR = 9.29, 95% CI = 1.72-61.78 in the very hard group) and ergonomic risks(OR = 4.50, 95% CI = 1.03-19.68). Shoulder (OR = 4.50, 95% CI = 1.03-19.68), hand and wrist (OR = 10.21, 95% CI = 1.02-102.20) are statistically significant risk factors in this study. But neck, Arm and elbow showed no significant relationship with WRMSDs symptoms (Table 3).

**Table 3:** **Odds ratios of factors related to upper extremities musculoskeletal symptoms by multiple logistic regression (n =166)**

**Factors**		**Neck**	**Shoulder**	**Arm/elbow**	**Hand/wrist**	**Upp. ext**
Age	30-39	1.00	1.00	1.00	1.00	1.00
	40-49	0.86(0.01-1.23)	3.10(0.48-19.91)	1.10(0.10-12.85)	2.12(0.18-24.29)	3.56(0.66-19.32)
	≥50	1.25(0.20-7.66)	3.80(0.59-24.50)	2.16(0.20-23.11)	4.44(0.41-47.7)	5.86(1.03-33.26)
BMI	<25	1.00	1.00	1.00	1.00	1.00
	≥25	2.12(0.38-11.81)	3.62(1.03-12.71)	4.38(0.82-23.50)	3.06(0.63-14.91)	3.26(1.28-13.53)
Working hours^*^	<9	1.00	1.00	1.00	1.00	1.00
	≥9	6.47(0.64-65.02)	1.41(0.39-5.15)	1.83(0.35-9.43)	3.30(0.52-21.16)	2.33(0.71-7.61)
Exercise^†^	YES	1.00	1.00	1.00	1.00	1.00
	No	2.77(0.40-19.05)	1.88(0.48-7.30)	2.16(0.37-12.72)	0.82(0.15-4.50)	2.37(1.24-12.59)
Burden	N to M‡	1.00	1.00	1.00	1.00	1.00
	Hard	6.30(0.54-73.74)	1.53(0.38-6.14)	0.58(0.13-9.26)	2.11(0.31-14.50)	1.63(0.46-5.81)
	Very hard	8.70(0.68-118.62)	9.29(1.72-51.78)	1.10(0.13-9.26)	3.67(0.43-31.32)	7.61(1.34-52.74)
Ergonomics	Low risk	1.00	1.00	1.00	1.00	1.00
	High risk	7.98(0.75-84.88)	4.50(1.03-19.68)	0.92(0.18-4.71)	10.21(1.02-102.20)	4.82(1.29-16.06)

*Working hours: hour ^†^Exercise Yes :> 3 times/week, >40 minutes/one time ^‡^N to M: None to moderate.

^†^Age, BMI, working hours, exercise, physical burden, ergonomics are adjusted.

WRMSDs symptoms, in the whole upper extremities, were higher in the 50 or older age group (OR = 5.86, 95% CI = 1.03-33.26), higher BMI group (OR = 3.26, 95% CI = 1.28-13.53), non-exercise group (OR = 2.37, 95% CI = 1.24-12.59), very hard physical burden group (OR = 7.6, 95% CI = 1.34-52.74), and high grade ergonomic risk group (OR = 4.82, 95% CI = 1.29-16.06).

## Discussion

The results of the study demonstrated that some occupational and non-occupational factors were related with WRMSDs symptoms in cameramen. The variables we choose were previously identified to have a close relationship with occupational musculoskeletal disorders in different jobs respectively [2-5]. Marital status is also one of the factors known to be able to influence on WRMSDs symptoms. But we excluded it at the level of variable selection because our subjects were almost married (92.8%) and we thought it would not make any statistical difference between those two groups. Not all of risk factors were found to be related with the symptoms in cameramen. We finally found out 5 factors that affect to WRMSDs symptoms in the job.

Working hours were statistically significant in univariable analysis. Working hours and break time proved to be a significant risk factor in previous studies [2,11,15,16]. But, in the logistic regression analysis, they turned out not to be significant in the relationship with WRMSDs symptoms. It is probably because working hours and break time of cameramen are mostly irregular compared to the other jobs because of their occupational characteristics.

BMI showed a significant association with increased score of musculoskeletal discomfort and occupational stress in a previous study, which implies that there must be some relationship between BMI and musculoskeletal disorders even though the subjects are different [17]. In the same vein, a study in 2013 also showed that exercise and BMI have influence to chronic musculoskeletal pain in a roundabout way [18]. Our study also showed the same result in the shoulder region where the highest symptom complaints occurred.

Symptom complaints did not show a linear relationship by the increase of tenure (Table 2). Musculoskeletal symptom complaints were relatively high in the first quartile and the fourth quartile. Such types of reversal are inevitable in cross-sectional studies, as increasing tenure and age can act as factors that lead to a reduction or change of work load.

The characteristics of ergonomic risk assessment were very clear. The most frequently checked scale among posture-related questions was ‘posture of the shoulder (located above chest level, 68.9%)’ , to followed by ‘flexion (≥20°)or extension(≥5°) of neck, 43.7%’ , ‘flexion (≥20°) or extension (≥30°) of wrist, 39.4%’. Those findings correspond with the fact that the positive rate of WRMSDs symptoms by parts of the body was turned out to be the highest in the shoulders (14.5%) followed by neck (7.8), hands and wrists (6.6). In logistic regression analysis, shoulder symptoms increased with BMI, physical burden, and ergonomic factors. For the sake of decrease in shoulder symptoms of cameramen, preventive management should focus on those factors.

Direct comparison of prevalence with the other jobs in previous studies is difficult because the definition of symptom varies depending on the study. But, we could compare the prevalence of musculoskeletal symptom by investigating other occupations in the same broadcasting station. According to our report, the percentage of WRMSDs high risk group in cameramen (19.3%) is unquestionably higher than the other occupations. The prevalence rates were 9.4% in technical post group, 6.2% in office job group, 2.1% in the other occupations (announcer, station security, janitor, etc.).

In a previous study about newsreel cameramen, carrying a heavy, mobile camera on the shoulder was found to be capable of causing suprascapular nerve entrapment and shoulder pain [19]. Thus, they asserted that those symptoms and findings should be considered as an occupational disorder, like meat-packer’s neuropathy. As shown in Table 2, the most frequently reported part of complaints is shoulder (14.5%), that is usually influenced when suprascapular nerve was entrapped by contact stress and pressure. So, there is possibility that some part of the complaints of shoulders was derived from suprascapular nerve entrapment by camera. Shoulder pads or some devices for decentering the weight could be helpful to prevent the entrapment from the weight and pressure of heavy mobile camera.

One of limitations of our study is that we have collected every data only from the questionnaire. Tenure and working hours were not based on the statement of service, which make a little difference from how long they actually have been worked. It must be done correctly in the further studies. Another limitation is derived from the structure of the study. We adapted a cross-sectional model which is not suitable to reveal causal relationship between musculoskeletal symptoms and risk factors. So, we cannot say that all risk factors we found were cause of the symptoms. Third, the number of subjects was not sufficient. The odds ratio and confidence interval for the variables were high and large compared to the previous studies [11,16]. Although the job of subjects were different (shipyard workers), the difference is thought to be come from the insufficiency of subjects. In a previous study, non-work relating and individual factors such as smoking and drinking, work-related psychosocial factors were found to be significant in upper extremity complaints [20,21]. But we did not take those non-work relating factors into account, which is the final limitation. With all those faults, this study is meaningful in the way that it was the first attempt to investigate risk factors and prevalence of musculoskeletal symptoms of cameramen. This job, until today little noticed by the public and researchers, will receive more attention by publication of the associated articles in the future.

## Conclusions

The most serious musculoskeletal disorders of male cameramen were shoulder pain. Ergonomic factors and physical burden were the most significant factors affecting WRMSDs in cameramen in this study. BMI and exercise were also somewhat related with complaints. Therefore, it is also necessary to make an effort to improve individual factors as well as work-related factors. Cameramen should be educated to be able to improve the ergonomic occupational environment and to set up preventive measures against the risk factors during work.
